# New entomopathogenic species in the Clavicipitaceae family (Hypocreales, Ascomycota) from the subtropical forests of Fujian, China

**DOI:** 10.3389/fmicb.2025.1532341

**Published:** 2025-03-03

**Authors:** Longbing Lin, Yongsheng Lin, Nemat O. Keyhani, Huili Pu, Jiao Yang, Chengjie Xiong, Junya Shang, Yuchen Mao, Lixia Yang, Minghai Zheng, Mengjia Zhu, Taichang Mu, Yi Li, Huiling Liang, Longfei Fan, Xiaoli Ma, Haixia Ma, Wen Xiong, Junzhi Qiu, Xiayu Guan

**Affiliations:** ^1^State Key Laboratory of Ecological Pest Control for Fujian and Taiwan Crops, College of Life Sciences, Fujian Agriculture and Forestry University, Fuzhou, China; ^2^Department of Biological Sciences, University of Illinois, Chicago, IL, United States; ^3^College of Food Science and Engineering, Yangzhou University, Yangzhou, China; ^4^Guangxi Institute of Botany, Chinese Academy of Sciences, Guilin, China; ^5^College of Plant Protection, Gansu Agricultural University, Lanzhou, China; ^6^College of Life Science and Technology, Xinjiang University, Urumqi, China; ^7^Institute of Tropical Bioscience and Biotechnology, Chinese Academy of Tropical Agricultural Sciences, Hainan Key Laboratory of Tropical Microbe Resources, Haikou, China; ^8^Forestry Diseases and Pests Control Station of Yongding District of Longyan City, Yongding, China; ^9^Key Laboratory of Ministry of Education for Genetics, Breeding and Multiple Utilization of Crops, College of Horticulture, Fujian Agriculture and Forestry University, Fuzhou, China

**Keywords:** entomopathogenic fungi, *Albacillium*, *Conoideocrella*, *Metarhizium*, *Neoaraneomyces*, Clavicipitaceae, new taxa

## Abstract

**Introduction:**

Entomopathogenic fungi play a crucial role in the ecological regulation of insect populations and can be exploited as a resource for pest control, sustainable agriculture, and natural products discovery. These fungi and their infected hosts are sometimes highly coveted as part of traditional medicine practices. Here, we sought to examine the biodiversity of entomogenous fungi in subtropical forests of China.

**Methods:**

Fungal-infected insect specimens were collected from various sites in Fujian Province, China, and purified isolates were obtained through laboratory cultivation and isolation techniques. Molecular characterization of specific target genomic loci was performed on the fungal isolates, and used for phylogenetic analyses using Bayesian inference and maximum likelihood methods to elucidate their taxonomic relationships. Microscopy was used to describe the morphological features of the isolates.

**Results:**

Through a comprehensive two-year survey of Fujian Province via multilocus molecular phylogenetic analysis targeting the nr*SSU*, nr*LSU*, *tef1-α*, *rpb1,* and *rpb2* loci of collected specimens, we identified three novel species within the Clavicipitaceae herein described as: *Albacillium fuzhouense* sp. nov., *Conoideocrella gongyashanensis* sp. nov. and *Neoaraneomyces wuyishanensis* sp. nov., as well as the recently recorded, *Metarhizium cicadae*. Each new species was also distinguished from its closest relatives by unique morphological characteristics.

**Discussion:**

These discoveries enrich our understanding of biodiversity within the Clavicipitaceae family and can contribute to the development of new pest control strategies and natural products discovery.

## 1 Introduction

Entomogenous fungi are pathogenic microorganisms that can infect and kill insect hosts (Litwin et al., 2020; Li et al., 2023). They play a key role in regulating the ecological balance of insect populations in a wide range of natural ecosystems, including forests, and have been exploited as a resource for pest biological control and sustainable agriculture. Various isolates of these fungi have been developed into promising green biopesticides (Pattemore et al., 2014; Hyde et al., 2019), contributing to biodiversity conservation by offering a more environmentally friendly approach to pest management. Currently, several species such as *Beauveria* Vuill., *Metarhizium* Sorokīn, and *Lecanicillium* W. Gams & Zare are widely used for applied biological control of agricultural pests (Mantzoukas and Eliopoulos, 2020). In addition, some isolates of plant-pathogenic fungi such as *Alternaria alternata* can also be pathogenic to insects (Paschapur et al., 2022). The family Clavicipitaceae (Ascomycota, Hypocreales) is distributed widely in nature and can be found in different trophic levels and organisms, e.g., plants, soil, insects, and other invertebrates (Sung et al., 2007; Steiner et al., 2011; Kepler et al., 2011; Thanakitpipattana et al., 2020). Recent studies have characterized the diversity of Clavicipitaceae into at least 53 genera with more than 750 species (Chen et al., 2022; Phull et al., 2022; Zhang et al., 2023). The morphology of Clavicipitaceae is usually characterized by cylindrical asci, thickened ascus apices, and filiform and multiseptate ascospores that are often disjoint at maturity (Rogerson, 1970; Sung et al., 2007). Clavicipitaceae fungi play an important role in plant protection and symbiotic evolution. They have diverse applications, including pest management and the treatment of conditions such as migraines, Alzheimer’s disease, and Parkinson’s disease through ergot alkaloids (Robinson and Panaccione, 2015; Florea et al., 2017). In addition, endophytes such as *Epichloë* enhance host plants’ stress resistance by producing bioactive compounds (Karpyn et al., 2017; Fernando et al., 2021).

The genus *Albacillium* was proposed by Ding et al. (2024) and first discovered in the forest litter of Northeast China. *Albacillium hingganense* was designated as the type species. Although *Albacillium* and *Chlorocillium* share a close relationship in phylogenetics, they exhibit differences in both morphological characteristics and phylogenetic placement determined by multilocus molecular data. *Albacillium* is characterized by conidia that are produced directly from phialides and arranged in slimy, globular clusters at the apices of the phialides. Currently, there is only one species within the genus *A. hingganense* (Ding et al., 2024).

*Metarhizium* is a genus that belongs to the family Clavicipitaceae and has rich morphological and ecological diversity (Brunner-Mendoza et al., 2019; Dakhel et al., 2020). Due to the incredible insect parasitizing ability of various *Metarhizium* members, certain species, e.g., *Metarhizium anisopliae*, *Metarhizium robertsii,* and *Metarhizium brunneum* have been used to develop eco-friendly commercial biological control agents (Meyling and Eilenberg, 2007; Zimmermann, 2007; Castrillo et al., 2011). *Metarhizium* species can be described by morphological characteristics such as mycelium/conidia (spore), conidia structure (size, arrangement, and production), and hyphal and other cell structure parameters (Sung et al., 2007). However, with the increasing number of species in this genus, morphological convergences can hinder species identification, with examples of cryptic species. In recent years, multigene phylogenetic studies have been able to exploit molecular approaches to better distinguish between various species of *Metarhizium* and its related genera (Luangsa-ard et al., 2017; Mongkolsamrit et al., 2020). *Metarhizium* sp. have been used in pest control worldwide for over 140 years (Nishi et al., 2013; González-Hernández et al., 2020), with continued discovery of diversity within *Metarhizium* being reported.

The genus *Torrubiella* Boud. was classified within Clavicipitaceae by Boudier in 1885 (Boudier, 1885). Fungi of this genus can parasitize a range of arthropods; however, spiders and scale insects appear to be particularly targeted hosts (Johnson et al., 2009). At present, there are 84 records of the genera in the Index (data from online service Index Fungorum http://www.indexfungorum.org; accessed on 15 September 2024). The majority of the species of *Torrubiella* are characterized by infected hosts wrapped in loose hyphae that generate conical, elongate perithecia and planar stromata (Johnson et al., 2009; Mongkolsamrit et al., 2016). *Torrubiella* is closely related to *Cordyceps* s. 1. but differs in morphology, although members have been subsequently divided into three distinct families (Clavicipitaceae, Cordycipitaceae, and Ophiocordycipitaceae) (Johnson et al., 2009). At the same time, a new genus, *Conoideocrella*, was proposed by Johnson et al. (2009), with *C. luteorostrata* (Zimm.) identified as the type species. Currently, *Conoideocrella* contains four species, all of which have a sexual morph similar to *Torrubiella* (Hywel-Jones, 1993; Mongkolsamrit et al., 2016; Wang et al., 2024). *C. krungchingensis* is parasitic on scale insects and was found on the undersides of fallen leaves in Thailand (Mongkolsamrit et al., 2016). In addition, *C. fenshuilingensis* appears to be distributed only in China (Wang et al., 2024).

The majority of the spider-pathogenic fungi are distributed in Cordycipitaceae and Ophiocordycipitaceae and include both generalist and specialist (essentially spider-specific) members, e.g., *Akanthomyces* Lebert, *Beauveria* Vuill., *Cordyceps*, *Gibellula* Cavara, *Hevansia* Luangsa-ard, *Hirsutella* Pat., *Hymenostilbe* Petch, *Purpureocillium* Luangsa-ard, and *Torrubiella* Boud (Chen et al., 2018; Chen et al., 2022; Joseph et al., 2024). The genus *Neoaraneomyces* is an araneopathogenic fungus first identified in the Clavicipitaceae family with *Neoaraneomyces araneicola* as the type species (Chen et al., 2022). *Neoaraneomyces* fungi produce white to gray mycelia completely covering the spider host. At present, there is only one species in this genus.

The biodiversity of entomopathogenic fungi in subtropical forests in China remains underexplored. Fujian Province in South-eastern China is highly forested (65.12%) and is environmentally neotropical. Its unique subtropical climate and biodiversity provide a rich habitat for diverse fungal species. During our survey assessing the diversity of entomogenous fungi across various regions of Fujian Province, we collected a variety of insect specimens exhibiting fungal infections. Combined molecular analyses using five genetic loci (nr*SSU*, nr*LSU*, *tef1-α*, *rpb1,* and *rpb2*) combined with detailed morphological characterization revealed three new species, one each within *Albacillium*, *Conoideocrella,* and *Neoaraneomyces*, as well as a new record of *Metarhizium cicadae* in China.

## 2 Materials and methods

### 2.1 Sample collection and isolation

Fungal-infected insect specimens were collected from understory vegetation, stones, and decomposing matter in mixed forests of Fuzhou City, the Gongyashan National Forest Park, Longqishan National Nature Reserve, and Wuyi Mountain National Nature Reserve in Fujian Province, China (Supplementary Figure S1), between September 2023 and June 2024, focusing on the summer months. Infected specimens were recorded and photographed in the field before collection and transport to the laboratory for further analyses. The specimens were stored in 50-mL sterile conical tubes containing a small amount of silica gel. To obtain purified isolates, stromata or synnemata from host bodies were taken in a sterile workbench, soaked in 75% alcohol for 30 s, and then washed twice with sterile water. After using sterilized filter paper to dry the tissue fragment, sample segments were inoculated onto potato dextrose agar (PDA: fresh potato chips 200 g/L, agar and dextrose 20 g/L, respectively) plates. After allowing for fungal mycelial growth (3–5 days), colony edges were used to inoculate fresh PDA plates until a pure culture was obtained by visual inspection. All characterized specimens have been preserved in the Fungarium (HMAS) at the Institute of Microbiology, Chinese Academy of Sciences. Characterized microbial strains have also been deposited with the China General Microbiological Culture Collection Center (CGMCC).

### 2.2 Morphological observations

A portion of the tissue from the pure culture (PDA plates) was picked and mounted in acid cotton blue on a slide. The microscopic morphological characteristics of the strains were observed using a Nikon Ni-U (Tokyo, Japan) compound microscope and a Nikon SMZ74 (Tokyo, Japan) stereomicroscope, and measurements were made using Digimizer image analysis software 6.4.0 (MedCalc Software Ltd., Belgium). Fungal cultures were also incubated on a PDA (25°C) for 2 weeks and subsequently photographed using a Canon EOS 6D Mark II (Tokyo, Japan) camera. Colony growth rates were calculated based on the methods of Liu and Hodge by taking measurements daily (Liu and Hodge, 2005).

### 2.3 DNA extraction, PCR, and sequencing

The total genomic DNA was extracted from the cultured mycelia using the Omega D3390 Fungal DNA Mini Kit (Guangzhou Feiyang Biological Engineering Co., LTD, China) following the manufacturer’s instructions. Five-gene loci including the nuclear ribosomal small subunit (nr*SSU*) (Wang et al., 2015), the nuclear ribosomal large subunit (nr*LSU*) (Vilgalys and Hester, 1990; Rehner and Samuels, 1994), translation elongation factor 1-*α* (*tef1-α*) (Bischof et al., 2006; Sung et al., 2007), and RNA polymerase II largest and second largest subunits (*rpb1* and *rpb2*) (Liu et al., 1999; Bischof et al., 2006; Sung et al., 2007) were targeted for PCR amplification and sequencing (primers given in Table 1, Sangon Biotech, Shanghai Co., Ltd). Each PCR amplification reaction consisted of 12.5 μL of 2 × Rapid Taq PCR Master Mix (Vazyme, Nanjing, China), 1 μL of each forward and reverse primer (10 μM), 1 μL of genomic DNA, and 9.5 μL of distilled deionized water (Sangon Bio Co., Ltd., Shanghai, China) to a total volume of 25 μL. PCR reactions were performed in a Bio-Rad thermal cycler (Hercules, CA, United States). The PCR products were purified and sequenced by Tsingke Biotech Co., Ltd. (Fuzhou, China). Sequences generated in this study have been submitted to GenBank (https://www.ncbi.nlm.nih.gov/genbank, under accession numbers as given in Table 2).

**Table 1:** The primer sequences used in this study.

Molecular marker	Primer	Sequence (5′–3′)	References
nr*SSU*	nr*SSU*-CoF	TCTCAAAGATTAAGCCATGC	Wang et al. (2015)
	nr*SSU*-CoR	TCACCAACGGAGACCTTG	
nr*LSU*	LR0R	GTACCCGCTGAACTTAAGC	Vilgalys and Hester (1990) Rehner and Samuels (1994)
	LR5	TCCTGAGGGAAACTTCG	
*tef1-α*	EF1α-EF	GCTCCYGGHCAYCGTGAYTTYAT	Bischof et al. (2006) Sung et al. (2007)
	EF1α-ER	ATGACACCRACRGCRACRGTYTG	
*rpb1*	RPB1-5′F	CAYCCWGGYTTYATCAAGAA	Bischof et al. (2006) Sung et al. (2007)
	RPB1-5′R	CCNGCDATNTCRTTRTCCATRTA	
*rpb2*	fRPB2-5F	GAYGAYMGWGATCAYTTYGG	Liu et al. (1999)
	fRPB2-7cR	CCCATWGCYTGCTTMCCCAT	

**Table 2:** Species, voucher information, locations, hosts, and corresponding GenBank accession numbers of the taxa used in this study.

Species	Voucher/Culture	Locations	Host/Substrate	GenBank accession number				
				nr*SSU*	nr*LSU*	*tef1-α*	*rpb1*	*rpb2*
***Albacillium fuzhouense***	**CGMCC3.27818**	**China**	**Coleoptera (larva)**	**PQ425616**	**PQ425618**	**PQ469143**	**PQ469145**	**PQ469147**
***Albacillium fuzhouense***	**CGMCC3.27815**	**China**	**Coleoptera (larva)**	**PQ425617**	**PQ425619**	**PQ469144**	**PQ469146**	**PQ469148**
*Albacillium hingganense*	CCTCC M 20232069	China	Forest litters	MN055707	OR740566	MN065771	OR769082	OR769081
*Aciculosporium oplismeni*	MAFF 246966	Japan	*Oplismenus undulatifolius*	-	LC571760	LC572040	-	LC572054
*Aciculosporium take*	MAFF 241224	Japan	*Phyllostachys pubescens*	-	LC571753	LC572034	-	LC572048
*Aciculosporium take*	TNS-F 60465	Japan	*Phyllostachys pubescens*	-	LC571756	LC572035	-	LC572049
*Aschersonia badia*	BCC 8105	Thailand	Hemiptera: scale insect	NG_062646	DQ518752	DQ522317	DQ522363	DQ522411
*Aschersonia placenta*	BCC 7869	Thailand	Hemiptera: scale insect	EF469121	EF469074	EF469056	EF469085	EF469104
*Atkinsonella hypoxylon*	B4728	USA	*Danthonia spicata*	-	-	KP689546	-	KP689514
*Balansia epichloe*	A.E.G. 96-15a	USA	Poaceae	EF468949	-	EF468743	EF468851	EF468908
*Balansia henningsiana*	GAM 16112	USA	*Panicum* sp. (Poaceae)	AY545723	AY545727	AY489610	AY489643	DQ522413
*Claviceps fusiformis*	ATCC 26019	-	Poaceae	DQ522539	U17402	DQ522320	DQ522366	-
*Claviceps purpurea*	GAM 12885	USA	Poaceae	-	AF543789	AF543778	-	DQ522417
*Claviceps purpurea*	S.A. cp11	-	Poaceae	EF469122	EF469075	EF469058	EF469087	EF469105
*Conoideocrella fenshuilingensis*	YHH CFFSL2310002	China	Hemiptera: scale insect	-	PP178583	PP776168	PP776158	-
*Conoideocrella fenshuilingensis*	YHH CFFSL2310003	China	Hemiptera: scale insect	-	PP178584	PP776169	PP776159	-
***Conoideocrella gongyashanensis***	**CGMCC3.28305**	**China**	**Dead spider**	**PQ286040**	**PQ278801**	**PQ301442**	**PQ316534**	**PQ334678**
***Conoideocrella gongyashanensis***	**CGMCC3.28306**	**China**	**Dead spider**	**PQ286041**	**PQ278802**	**PQ301443**	**PQ316535**	**PQ334679**
*Conoideocrella krungchingensis*	BCC 36100	Thailand	Hemiptera: scale insect	-	KJ435080	KJ435097	-	-
*Conoideocrella krungchingensis*	BCC 36101	Thailand	Hemiptera: scale insect	-	KJ435081	KJ435098	-	-
*Conoideocrella luteorostrata*	NHJ 11343	Thailand	Hemiptera: scale insect	EF468995	EF468850	EF468801	EF468906	-
*Conoideocrella luteorostrata*	NHJ 12516	Thailand	Hemiptera: scale insect	EF468994	EF468849	EF468800	EF468905	EF468946
*Conoideocrella tenuis*	NHJ 6293	Thailand	Hemiptera: scale insect	EU369112	EU369044	EU369029	EU369068	EU369087
*Conoideocrella tenuis*	NHJ 345.01	Thailand	Hemiptera: scale insect	EU369111	EU369045	EU369030	-	EU369088
*Epichloe elymi*	C. Schardl 760	USA	-	-	AY986924	AY986951	DQ000352	-
*Epichloe typhina*	ATCC 56429	USA	Fragaria sp. (Rosaceae)	U32405	U17396	AF543777	AY489653	DQ522440
*Metarhiziopsis microspora*	CEHS133a	USA	*Fiorinia externa*	-	EF464571	-	-	-
*Metarhiziopsis microspora*	INEHS133a	USA	*Fiorinia externa*	-	EF464572	-	-	-
*Metarhizium acridum*	ARSEF 7486	Niger	Orthoptera	-	-	EU248845	EU248897	EU248925
*Metarhizium album*	ARSEF 2082	Indonesia	Hemiptera	DQ522560	DQ518775	DQ522352	DQ522398	DQ522452
*Metarhizium alvesii*	CG 1123	Brazil	Soil	-	-	KY007614	KY007612	KY007613
*Metarhizium anisopliae*	ARSEF 7487	Ethiopia	Orthoptera	-	-	DQ463996	DQ468355	DQ468370
*Metarhizium anisopliae*	CBS 130.71	Ukraine	*Avena sativa*	MT078868	MT078853	MT078845	MT078861	MT078918
*Metarhizium argentinense*	CEP424	Argentina	Blaberidae: Epilamprinae	-	-	MF966624	MF966625	MF966626
*Metarhizium atrovirens*	TNM-F 10184	Japan	Coleoptera	JF415950	JF415966	-	JN049884	-
*Metarhizium baoshanense*	CCTCC M 2016589	China	Soil	KY264177	KY264174	KY264169	KY264180	KY264183
*Metarhizium baoshanense*	BUM 63.4	China	Soil	KY264178	KY264175	KY264170	KY264181	KY264184
*Metarhizium bibionidarum*	NBRC 112661	China	Diptera: Bibionidae	-	-	LC126076	LC125908	LC125924
*Metarhizium bibionidarum*	CBS 648.67	China	Diptera: Bibionidae	-	-	LC126075	LC125907	LC125923
*Metarhizium biotecense*	BCC 51812	Thailand	Hemiptera: Delphacidae	MN781937	MN781838	MN781693	MN781745	MN781792
*Metarhizium biotecense*	BCC 51813	Thailand	Hemiptera: Delphacidae	MN781938	MN781839	MN781694	MN781746	MN781793
*Metarhizium blattodeae*	MY00896	Thailand	Blattodea	HQ165657	HQ165719	HQ165678	HQ165739	HQ165638
*Metarhizium brachyspermum*	CM1	Japan	Coleoptera	-	LC469749	LC469751	-	-
*Metarhizium brasiliense*	ARSEF 2948	Brazil	Hemiptera	-	-	KJ398809	KJ398620	-
*Metarhizium brittlebankisoides*	Hn1	China	Coleoptera	-	-	AB778556	AB778555	AB778554
*Metarhizium brunneum*	ARSEF 2107	USA	Coleoptera	-	-	EU248855	EU248907	EU248935
*Metarhizium brunneum*	CBS 316.51	USA	Wireworm of *Agriotes* sp.	MT078875	MT078860	MT078852	-	MT078924
*Metarhizium campsosterni*	BUM 10	China	Soil	MH143832	MH143815	MH143849	MH143864	MH143879
*Metarhizium candelabrum*	BCC 29224	Thailand	Hemiptera: leafhopper	MN781952	MN781853	MN781708	MN781755	MN781804
*Metarhizium cercopidarum*	BCC 31660	Thailand	Hemiptera: leafhopper	MN781953	MN781854	MN781709	MN781756	MN781805
*Metarhizium chaiyaphumense*	BCC 78198	Thailand	Hemiptera: Cicadidae	KX369596	KX369593	KX369592	KX369594	KX369595
*Metarhizium cicadae*	BCC 48696	Thailand	Hemiptera: Cicadidae	MN781948	MN781848	MN781703	-	MN781800
*Metarhizium cicadae*	BCC 48881	Thailand	Hemiptera: Cicadidae	MN781949	MN781849	MN781704	MN781752	-
***Metarhizium cicadae***	**CGMCC3.28301**	**China**	**Hemiptera: Cicadidae**	**PQ303697**	**PQ278797**	**PQ301438**	**PQ316532**	**PQ334674**
***Metarhizium cicadae***	**CGMCC3.28302**	**China**	**Hemiptera: Cicadidae**	**PQ303698**	**PQ278798**	**PQ301439**	**PQ316533**	**PQ334675**
***Metarhizium cicadae***	**CGMCC3.28303**	**China**	**Hemiptera: Cicadidae**	**PQ303699**	**PQ278799**	**PQ301440**	**PQ316531**	**PQ334676**
***Metarhizium cicadae***	**CGMCC3.28304**	**China**	**Hemiptera: Cicadidae**	**PQ303700**	**PQ278800**	**PQ301441**	**PQ316530**	**PQ334677**
*Metarhizium clavatum*	BCC 84543	Thailand	Coleoptera (larva)	-	MN781834	MN781689	MN781741	MN781789
*Metarhizium clavatum*	BCC 84558	Thailand	Coleoptera (larva)	-	MN781835	MN781690	MN781742	-
*Metarhizium culicidarum*	BCC 2673	Thailand	Diptera: Culicidae	MN781950	MN781851	MN781706	MN781753	MN781802
*Metarhizium culicidarum*	BCC 7600	Thailand	Diptera: Culicidae	MN781951	MN781852	MN781707	MN781754	MN781803
*Metarhizium culicidarum*	BCC 7625	Thailand	Diptera: Culicidae	-	MN781850	MN781705	-	MN781801
*Metarhizium cylindrosporum*	RCEF 3632	China	Hemiptera: Cicadidae	JF415964	JF415987	JF416022	-	-
*Metarhizium cylindrosporum*	TNS-F 16371	Japan	Hemiptera: Cicadidae	JF415963	JF415986	JF416027	JN049902	-
*Metarhizium dendrolimatilis*	GZAC-IFR1006	China	Lepidoptera	-	-	KT166031	KT961694	KT166032
*Metarhizium dianzhongense*	KUNCC 10810	China	Coleoptera: Scarabaeidae	-	PP256152	PP328485	PP294694	PP314012
*Metarhizium dianzhongense*	KUNCC 10811	China	Coleoptera: Scarabaeidae	-	PP256150	PP328483	PP294692	PP314010
*Metarhizium eburneum*	BCC 79252	Thailand	Lepidoptera (pupa)	-	MN781829	MN781682	MN781736	-
*Metarhizium eburneum*	BCC 79267	Thailand	Lepidoptera (pupa)	-	MN781826	-	MN781735	-
*Metarhizium ellipsoideum*	BCC 12847	Thailand	Hemiptera (adult)	MN781959	MN781860	MN781715	MN781761	MN781810
*Metarhizium ellipsoideum*	BCC 49285	Thailand	Hemiptera (adult)	MN781957	MN781858	MN781713	MN781759	MN781808
*Metarhizium ellipsoideum*	BCC 53509	Thailand	Hemiptera (adult)	MN781958	MN781859	MN781714	MN781760	MN781809
*Metarhizium flavoviride*	CBS 125.65	USA	Soil	MT078869	MT078854	MT078846	MT078862	MT078919
*Metarhizium flavoviride*	CBS 218.56	Czech Republic	Coleoptera	-	MH869139	KJ398787	KJ398598	-
*Metarhizium flavum*	BCC 90870	Thailand	Coleoptera (larva)	MN781965	MN781874	MN781731	MN781776	MN781822
*Metarhizium flavum*	BCC 90874	Thailand	Coleoptera (larva)	MN781966	MN781875	MN781732	MN781777	MN781823
*Metarhizium frigidum*	ARSEF 4124	Australia	Coleoptera	-	-	DQ464002	DQ468361	DQ468376
*Metarhizium fusoideum*	BCC 28246	Thailand	Lepidoptera	MN781944	MN781844	MN781699	MN781749	MN781796
*Metarhizium fusoideum*	BCC 41242	Thailand	Psocoptera	MN781942	MN781825	MN781679	-	MN781780
*Metarhizium fusoideum*	BCC 53130	Thailand	Psocoptera	MN781943	MN781843	MN781698	-	MN781795
*Metarhizium gaoligongense*	CCTCC M 2016588	China	Soil	KY087812	KY087816	KY087820	KY087824	KY087826
*Metarhizium gaoligongense*	BUM 3.5	China	Soil	KY087810	KY087814	KY087818	KY087822	-
*Metarhizium globosum*	ARSEF 2596	India	Lepidoptera	-	-	EU248846	EU248898	EU248926
*Metarhizium granulomatis*	UAMH 11028	Denmark	*Chamaeleo calyptratus*	HM635076	HM195304	KJ398781	-	-
*Metarhizium granulomatis*	UAMH 11176	Denmark	*Chamaeleo calyptratus*	-	HM635078	KJ398782	KJ398593	-
*Metarhizium gryllidicola*	BCC 37918	Thailand	Orthoptera: Gryllidae	MN781935	MN781836	MN781691	MN781743	MN781790
*Metarhizium gryllidicola*	BCC 82988	Thailand	Orthoptera: Gryllidae	MK632117	MK632091	MK632062	MK632166	MK632143
*Metarhizium guizhouense*	ARSEF 6238	China	Lepidoptera	-	-	EU248857	EU248909	EU248937
*Metarhizium guizhouense*	CBS 258.90	China	Lepidoptera	-	MH873894	EU248862	EU248914	EU248942
*Metarhizium huainamdangense*	BCC 32190	Thailand	Hemiptera: leafhopper	MN781954	MN781855	MN781710	MN781757	-
*Metarhizium huainamdangense*	BCC 44270	Thailand	Hemiptera: leafhopper	MN781956	MN781857	MN781712	-	MN781807
*Metarhizium humberi*	IP 46	Brazil	Soil	-	-	MH837574	MH837556	MH837565
*Metarhizium indigoticum*	TNS-F 18553	Japan	Lepidoptera	JF415953	JF415968	JF416010	JN049886	JF415992
*Metarhizium indigoticum*	TNS-F 18554	Japan	Lepidoptera	JF415952	JF415969	JF416011	JN049887	JF415993
*Metarhizium kalasinense*	BCC 53582	Thailand	Coleoptera (larva)	KC011175	KC011183	KC011189	-	-
*Metarhizium koreanum*	BCC 27998	Thailand	Hemiptera: Fulgoromorpha	MN781945	MN781845	MN781700	-	MN781797
*Metarhizium koreanum*	BCC 30455	Thailand	Hemiptera: Fulgoromorpha	MN781946	MN781846	MN781701	MN781750	MN781798
*Metarhizium lepidiotae*	ARSEF 7488	Australia	Coleoptera	-	-	EU248865	EU248917	-
*Metarhizium macrosemiae*	RCEF 6696	China	Hemiptera: Cicadidae	MW718233	MW718259	MW723113	MW723144	MW723164
*Metarhizium macrosemiae*	RCEF 6729	China	Hemiptera: Cicadidae	MW718234	MW718260	MW723115	MW723145	MW723165
*Metarhizium majus*	ARSEF 1015	Japan	Lepidoptera	-	-	EU248866	EU248918	EU248946
*Metarhizium majus*	ARSEF 1914	Philippines	Coleoptera	-	-	EU248868	EU248920	EU248948
*Metarhizium megapomponiae*	BCC 25100	Thailand	Hemiptera: Megopomponia	MN781947	MN781847	MN781702	MN781751	MN781799
*Metarhizium minus*	ARSEF 2037	Philippines	Hemiptera	AF339580	AF339531	DQ522353	DQ522400	DQ522454
*Metarhizium minus*	ARSEF 1099	Philippines	Hemiptera	-	-	KJ398799	KJ398608	KJ398706
*Metarhizium niveum*	BCC 52400	Thailand	Hemiptera: Cicadidae	MN781933	MN781832	MN781685	-	MN781785
*Metarhizium nornnoi*	BCC 19364	Thailand	Lepidoptera (larva)	MN781940	MN781841	MN781696	MN781747	-
*Metarhizium nornnoi*	BCC 25948	Thailand	Coleoptera (adult beetle)	MN781941	MN781842	MN781697	MN781748	-
*Metarhizium novozealandicum*	ARSEF 4661	Australia	Soil	-	-	KJ398811	KJ398622	KJ398720
*Metarhizium novozealandicum*	ARSEF 4674	Australia	Soil	-	-	KJ398812	KJ398623	KJ398721
*Metarhizium ovoidosporum*	BCC 29223	Thailand	Hemiptera: Cercopidae	MN781960	MN781861	MN781716	MN781762	-
*Metarhizium ovoidosporum*	BCC 32600	Thailand	Hemiptera: Eurybrachidae	MN781961	MN781862	MN781717	MN781763	-
*Metarhizium ovoidosporum*	BCC 7634	Thailand	Hemiptera (adult)	MN781962	MN781863	MN781718	MN781764	MN781811
*Metarhizium owariense*	NBRC 33258	Japan	Hemiptera	HQ165669	HQ165730	HQ165689	HQ165747	-
*Metarhizium pemphigi*	ARSEF 6569	UK	Hemiptera	-	-	KJ398813	KJ398624	DQ468378
*Metarhizium pemphigi*	ARSEF 7491	UK	Hemiptera	-	-	KJ398819	KJ398629	DQ468379
*Metarhizium phasmatodeae*	BCC 2841	Thailand	Orthoptera: Phasmatodea	MN781931	MN781828	MN781681	MN781738	MN781782
*Metarhizium phasmatodeae*	BCC 49272	Thailand	Orthoptera: Phasmatodea	MK632119	MK632093	MK632064	-	MK632145
*Metarhizium phuwiangense*	BCC 78206	Thailand	Coleoptera (adult)	-	-	MN781719	MN781765	MN781812
*Metarhizium phuwiangense*	BCC 85069	Thailand	Coleoptera (adult)	-	MN781865	MN781721	MN781767	MN781814
*Metarhizium pinghaense*	CBS 257.90	China	Coleoptera	-	MH873893	EU248850	EU248902	-
*Metarhizium pinghaense*	BCC 47950	Thailand	Lepidoptera	KC011172	KC011180	KC011186	KC011184	-
*Metarhizium pinghaense*	BCC 47979	Thailand	Lepidoptera	KC011173	KC011181	KC011187	KC011185	-
*Metarhizium pseudoatrovirens*	TNS-F 16380	USA	Coleoptera	-	JF415977	-	JN049893	JF415997
*Metarhizium purpureogenum*	MAFF 243305	Japan	Soil	-	AB700552	LC126078	LC125913	LC125920
*Metarhizium purpureogenum*	MAFF 244762	Japan	Soil	-	-	LC126079	LC125911	LC125922
*Metarhizium purpureonigrum*	BCC 89247	Thailand	Coleoptera (larva)	-	-	MN781725	MN781771	MN781817
*Metarhizium purpureonigrum*	BCC 89249	Thailand	Coleoptera (larva)	MN781963	MN781869	MN781726	MN781772	MN781818
*Metarhizium purpureum*	BCC 82642	Thailand	Coleoptera (larva)	-	MN781867	MN781723	MN781769	MN781816
*Metarhizium purpureum*	BCC 83548	Thailand	Coleoptera (larva)	-	MN781868	MN781724	MN781770	-
*Metarhizium putuoense*	HMAS 285457	China	Coleoptera (larva)	OQ981977	OQ981970	OQ980403	OQ980411	-
*Metarhizium putuoense*	HMAS 285458	China	Coleoptera (larva)	OQ981978	OQ981971	OQ980404	OQ980412	-
*Metarhizium reniforme*	IndGH96	Philippines	–	HQ165670	HQ165732	-	-	HQ165649
*Metarhizium reniforme*	ARSEF 429	Philippines	Orthoptera: Tettigoniidae	HQ165671	HQ165733	HQ165690	-	HQ165650
*Metarhizium rileyi*	CBS 806.71	USA	*Trichoplusia ni*	AY526491	MH872111	EF468787	EF468893	EF468937
*Metarhizium rileyi*	NBRC 8560	Japan	Lepidoptera	HQ165667	HQ165729	HQ165688	-	-
*Metarhizium robertsii*	ARSEF 4739	Australia	Soil	-	-	EU248848	EU248900	EU248928
*Metarhizium samlanense*	BCC 17091	Thailand	Hemiptera (adult)	HQ165665	HQ165727	HQ165686	-	HQ165646
*Metarhizium samlanense*	BCC 39752	Thailand	Hemiptera (adult)	MN781939	MN781840	MN781695	-	MN781794
*Metarhizium sulphureum*	BCC 36585	Thailand	Lepidoptera (larva)	-	-	MN781686	-	MN781786
*Metarhizium sulphureum*	BCC 39045	Thailand	Lepidoptera (larva)	MK632120	MK632095	MK632066	-	MK632147
*Metarhizium sulphureum*	BCC 36592	Thailand	Lepidoptera (larva)	-	-	MN781687	-	MN781787
*Metarhizium takense*	BCC 30939	Thailand	Hemiptera	HQ165659	HQ165721	HQ165680	HQ165741	HQ165640
*Metarhizium takense*	BCC 30934	Thailand	Hemiptera	HQ165658	HQ165720	HQ165679	HQ165740	HQ165639
*Metarhizium viride*	CBS 659.71	-	*Chamaeleo lateralis*	HQ165673	HQ165735	HQ165692	-	HQ165652
*Metarhizium viridulum*	BCC 36261	Thailand	Hemiptera: Cicadidae	MN781930	MN781827	MN781680	MN781737	MN781781
*Metarhizium viridulum*	ARSEF 6927	Taiwan	Hemiptera	-	-	KJ398815	KJ398626	-
*Moelleriella phyllogena*	P.C. 555	Bolivia	Scale insects and whiteflies	-	EU392610	EU392674	EU392726	-
*Moelleriella phyllogena*	J.B. 130	Panama	Scale insects and whiteflies	-	EU392608	EU392672	EU392724	-
*Moelleriella umbospora*	P.C. 461	Mexico	Scale insects or whiteflies	-	EU392628	EU392688	EU392740	-
*Myriogenospora atramentosa*	A.E.G. 96–32	-	Plant	AY489701	AY489733	AY489628	AY489665	DQ522455
*Neoaraneomyces araneicola*	DY101711	China	Spider	-	MW730609	MW753033	MW753024	MW753026
*Neoaraneomyces araneicola*	DY101712	China	Spider	-	MW730610	MW753034	-	MW753027
***Neoaraneomyces wuyishanensis***	**CGMCC3.28307**	**China**	**Spider**	**PQ286042**	**PQ278803**	**PQ301444**	**PQ316536**	**PQ334680**
***Neoaraneomyces wuyishanensis***	**CGMCC3.28308**	**China**	**Spider**	**PQ286043**	**PQ278804**	**PQ301445**	**PQ316537**	**PQ334681**
*Nigelia aurantiaca*	BCC 13019	Thailand	Lepidoptera (larva)	GU979939	GU979948	GU979957	GU979966	GU979971
*Nigelia martiale*	EFCC 6863	Republic of Korea	Lepidoptera	-	JF415975	JF416016	-	JF415995
*Orbiocrella petchii*	NHJ 6240	Thailand	Hemiptera: scale insect	EU369103	EU369038	EU369022	EU369060	EU369082
*Orbiocrella petchii*	NHJ 6209	Thailand	Hemiptera: scale insect	EU369104	EU369039	EU369023	EU369061	EU369081
*Parametarhizium changbaiense*	SGSF125	China	Forest litter	MN590231	MN589994	MN908589	MN917168	-
*Parametarhizium hingganense*	SGSF355	China	Forest litter	MN055706	MN061635	MN065770	MN917170	MT939494
*Paraneoaraneomyces sinensis*	ZY 22.006	China	Soil	OQ709248	OQ709260	OQ719626	-	OQ719621
*Paraneoaraneomyces sinensis*	ZY 22.007	China	Soil	OQ709249	OQ709261	OQ719627	-	OQ719622
*Paraneoaraneomyces sinensis*	ZY 22.008	China	Soil	OQ709250	OQ709262	OQ719628	-	OQ719623
*Pleurocordyceps aurantiaca*	MFLUCC 17–2,113	China	*Ophiocordyceps barnesii*	MG136904	MG136910	MG136875	MG136866	MG136870
*Pleurocordyceps marginaliradians*	MFLU 17–1,582	China	Lepidoptera: Cossidae	MG136908	MG136914	MG136878	MG136869	MG271931
*Samuelsia chalalensis*	P.C. 560	Bolivia	Bamboo	-	EU392637	EU392691	EU392743	-
*Samuelsia mundiveteris*	BCC 40021	Thailand	Hemiptera: Scale insect nymphs	-	GU552152	GU552145	-	-
*Samuelsia rufobrunnea*	P.C. 613	Bolivia	Leaves	-	AY986918	AY986944	DQ000345	-
*Shimizuomyces paradoxus*	EFCC 6279	Republic of Korea	Smilax sieboldii	EF469131	EF469084	EF469071	EF469100	EF469117
*Shimizuomyces paradoxus*	EFCC 6564	Republic of Korea	Smilax sieboldii	EF469130	EF469083	EF469072	EF469101	EF469118
*Yosiokobayasia kusanagiensis*	TNS-F 18494	Japan	Coleoptera	JF415954	JF415972	JF416014	JN049890	-

Data produced in this study are presented in boldface.

### 2.4 Phylogenetic analyses

Phylogenetic analyses were performed based on the five genetic loci examined (nr*SSU*, nr*LSU*, *tef1-α*, *rpb1,* and *rpb2*) and corresponding sequences from GenBank. GenBank accession numbers of comparative taxa used are listed in Table 2. MAFFT v. 7 online website (http://mafft.cbrc.jp/alignment/server/, accessed on 17 September 2024) was used to align all the sequences, and then sequences were manually edited using BioEdit v.7.2.6.1 (He et al., 2022) and MEGA v.7.0 (Kumar et al., 2016) as needed. The aligned loci sequences were concatenated using Phylosuite v1.2.2 (Zhang et al., 2020). Based on five-gene combination datasets, phylogenetic analyses were performed using Bayesian inference (BI) and maximum likelihood (ML). The ML and Bayesian analyses were conducted using IQ-tree v.2.1.3 (Nguyen et al., 2015) and MrBayes v.3.2.2 (Ronquist et al., 2012), respectively. Four simultaneous Markov chains were run for 2,500,000 generations, and trees were sampled with a frequency of every 100th generation. The initial 25% of sampled trees were discarded as burn-in. The clades support value over 0.9 in BI and greater than 70% in ML were considered significantly supported. Phylogenetic trees were viewed using the ITOL online service^1^ and FigTree v.1.4.3 and polished using Adobe Illustrator 2023.

## 3 Results

### 3.1 Sample collection and processing

Approximately 40 specimens were collected from four distinct locations in Fujian Province: Gongya Mountain National Forest Park, Longqishan National Nature Reserve, Wuyi Mountain National Nature Reserve, and Fujian Agriculture and Forestry University. More than 35 samples were purified into single colonies, and 12 strains showed unique morphological signatures. Genomic DNA was extracted from these isolates, and selected genetic loci were sequenced, as detailed in the Methods section. Of the 12 specimens examined, 3 were identified as novel isolates (with 1 being a first description in China), as further detailed below.

### 3.2 Sequencing and phylogenetic analyses

**Figure 1:**
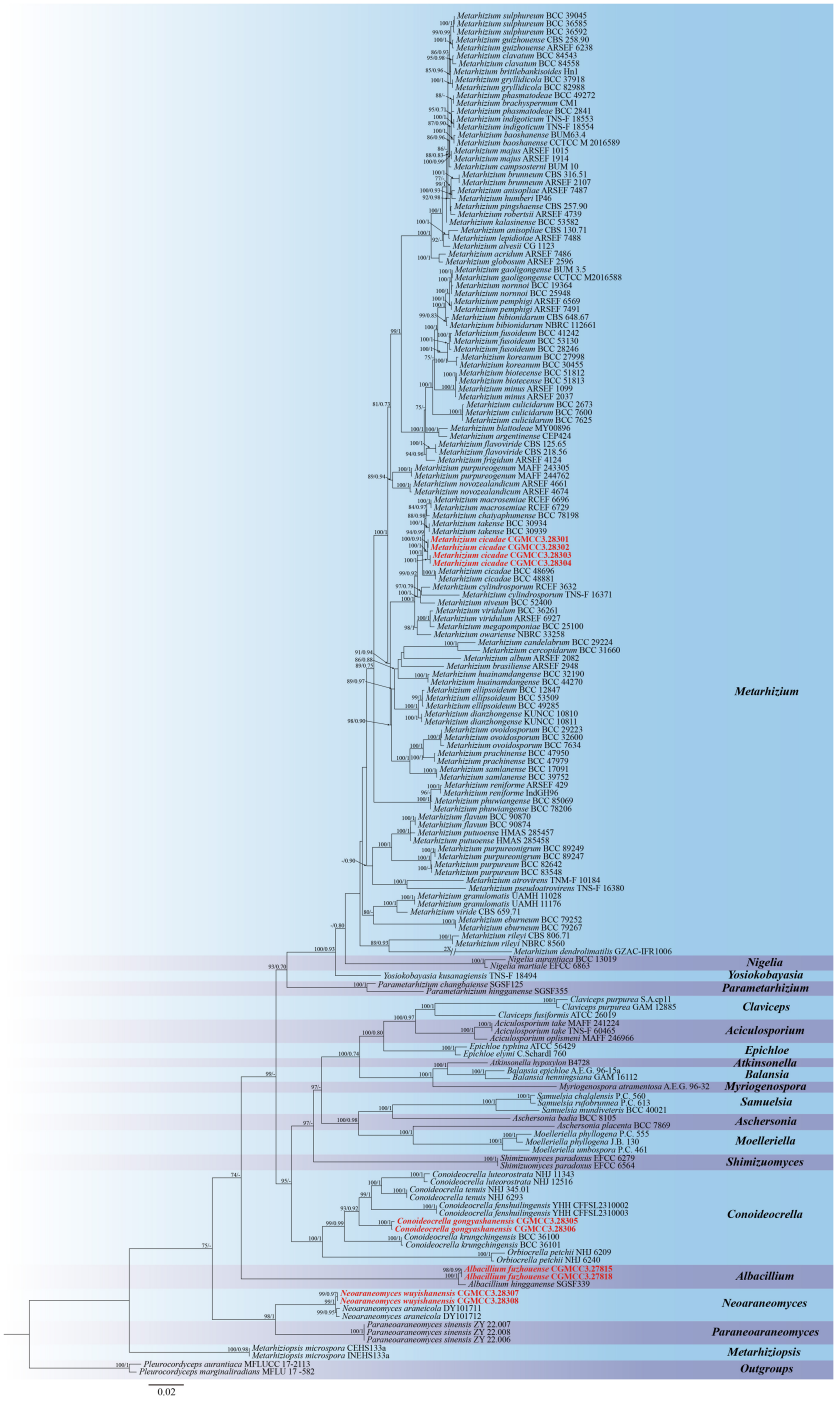
Phylogenetic analysis of Clavicipitaceae based on maximum likelihood (ML) and Bayesian inference (BI) using five-gene combination (nr*SSU*, nr*LSU*, *tef1-α*, *rpb1,* and *rpb2*). The ML bootstrap proportions (left) and BI posterior probabilities (right) greater than 70% and 0.7 were shown above brunches. Strains in both bold and red are generated in this study.

Five genetic loci (nr*SSU*, nr*LSU*, *tef1-α*, *rpb1,* and *rpb2*) were used to construct both ML and BI trees for molecular identification supporting new species descriptions in this study. *Pleurocordyceps aurantiaca* (MFLUCC 17–2,113) and *Pleurocordyceps marginaliradians* (MFLU 17–1,582) were selected as outgroups in the phylogenetic reconstruction of Clavicipitaceae. The total length of the concatenated sequences was 4,508 bp (nr*SSU*: 1–1,042 bp; nr*LSU*: 1043–1932 bp; *tef1-α*: 1933–2,829 bp; *rpb1*: 2830–3,518 bp, and *rpb2*: 3519–4,508 bp), with analyses including members from 172 taxa from 20 genera. The phylogenetic trees derived from both BI and ML analyses exhibited similar topological structures and indicated three new species and one newly recorded species within Clavicipitaceae as being strongly supported (Figure 1). These molecular analyses revealed that (1) the newly described *C. gongyashanensis* sp. nov. was closely related to *C. fenshuilingensis* and *C. krungchingensis* with high support value (ML-BS: 93%, BI-PP: 0.92), and (2) that two specimens were identical and described in this study as *Albacillium fuzhouense*, within *Albacillium* with strong support (BP = 100% and PP = 1). Analyses of four specimens that grouped within the *Metarhizium* clade indicated a monophyletic cluster that could be assigned to *M. cicadae* (BP = 94% and PP = 0.99). In addition, two specimens are described in this study as a new species, *N. wuyishanensis*, which is closely related to its syntopic species *N. araneicola*, forming a collective group (BP: 99%, PP: 1).

### 3.3 Taxonomy

**Figure 2:**
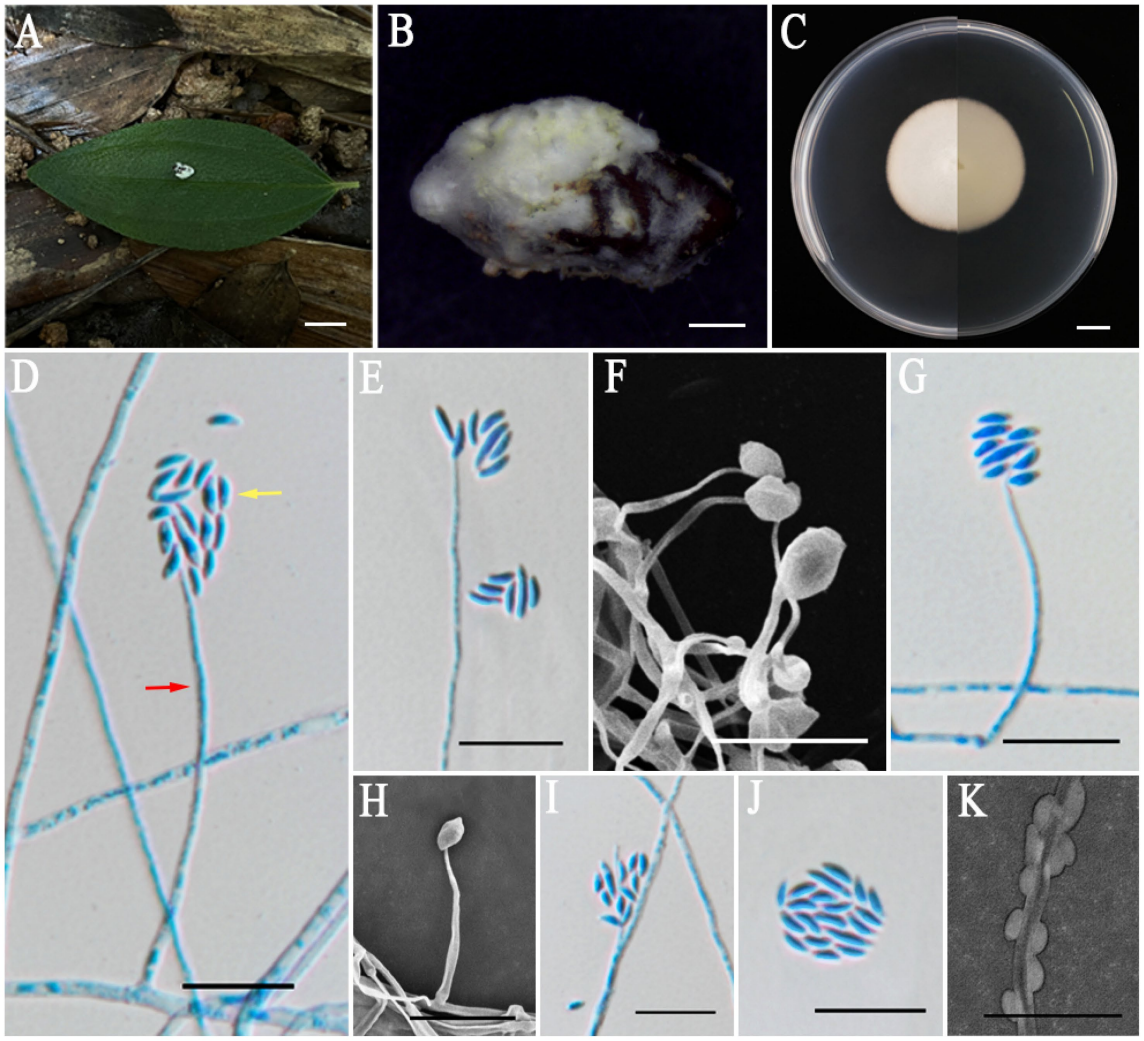
*Albacillium fuzhouense* (holotype HMAS353206). **(A,B)** Larva of Coleoptera infected by *A. fuzhouense*. **(C)** Colony characteristics on PDA for 14 days. **(D–K)** Conidiophores (*red arrow*) and conidia (*yellow arrow*) on PDA. Scale bars: **(A,C)** 5 mm; **(B)** 1 mm; **(D–K)** 10 μm.

*Albacillium fuzhouense* L. B. Lin and J. Z. Qiu, sp. nov. (Figure 2).

MycoBank No.: MB856115.

Etymology: Named after Fuzhou City, where the specimen was originally found.

Holotype: China, Fujian Province, Fuzhou City, 119°14′32″ E, 26°4′54″ N, alt. 62 m, found on a larva of Coleoptera attached to the leaf litter in a bamboo garden, 19 June 2024, J. Z. Qiu and L. B. Lin (HMAS353206, holotype; ex-holotype living culture CGMCC3.27818).

Sexual morph: Undetermined.

Asexual morph: Larvae (Coleoptera) covered by white and cottony mycelium, producing white and powdery conidia. Colonies grew well on PDA medium at a relatively constant rate, attaining a diameter of 38–39 mm after 14 days of cultivation at 25°C, with white, felty, dense mycelium, an entire margin, and reverse light yellow. Hyphae were hyaline, smooth-walled, septate, 1.1–2.1 μm wide. Phialides directly originated from aerial hyphae, lanceolate, solitary, hyaline, with a cylindrical base tapering to apex, 13.6–81.5 × 0.8–1.3 μm, mean = 36.9 × 1.1 μm. Conidia were fusiform, ellipsoidal and slightly curved, hyaline, smooth, one-celled, arranged either singly or in mucilaginous spherical clusters at the tips of the phialides, 2.4–6.7 × 1.0–1.4 μm, mean = 4.2 × 1.2 μm.

Habitat: Parasitic on larvae of Coleoptera attached to the leaf litter.

Distribution: China, Fujian Province, Fuzhou City, Jianxin Town.

Other materials examined: China, Fujian Province, Fuzhou City, 119°14′32″ E, 26°4′54″ N, alt. 1,084 m, found on a larva of Coleoptera attached to the leaf litter in a bamboo garden, 19 June 2024, J. Z. Qiu and L. B. Lin (HMAS353207, paratype; ex-paratype living culture CGMCC3.27815).

Notes: The five-gene multilocus phylogenetic analysis revealed that the two samples of *A. fuzhouense* clustered with *A. hingganense* with strong support (BP: 100%, PP: 1). Then, the nucleotide sequence comparison of *rpb2* showed a 2.1% (23/1091) difference between *A. fuzhouense* and its related species. Morphologically, *A. fuzhouense* was similar in shape to its close relatives, yet is distinguished by possessing longer phialides (13.6–81.5 × 0.8–1.3 vs. 8.6–60.1 × 0.9–1.5 μm) and larger conidia (2.4–6.7 × 1.0–1.4 vs. 2.0–4.3 × 0.8–1.3 μm) compared to those of *A. hingganense*. In addition, the colonies on PDA of *A. fuzhouense* grew faster than *A. hingganense,* and the textures of the two are different. *A. hingganense* is velvet, while *A. fuzhouense* is more of a felty texture. Moreover, there existed a notable divergence in the host organisms and origins between these two species. The newly found species of the present study were parasitic on the larvae of Coleoptera and were isolated from the Fujian subtropical forest, whereas *A. hingganense* was isolated from forest litters in a cold temperate climatic zone (Table 3).

**Table 3:** Comparison between the species generated in this study and their closely related species.

Species	Country	Host	Phialides (μm)	Conidia (μm)	Colony	References
***Albacillium fuzhouense***	**China**	**Coleoptera (larva)**	**Lanceolate, solitary, with a cylindrical base tapering to apex, 13.6–81.5** **×** **0.8–1.3**	**Fusiform, ellipsoidal, and slightly curved,** **2.4–6.7 × 1.0–1.4**	**White**	**This study**
*Albacillium hingganense*	China	Forest litters (fallen leaves and soil)	Short to slender, straight to flexuous, tapering toward the apex, 8.6–60.1 × 0.9–1.5	Ellipsoidal to cymbiform, straight to slightly curved, 2.0–4.3 × 0.8–1.3	White	Ding et al. (2024)
*Conoideocrella fenshuilingensis*	China	Scale insects	-	-	-	Wang et al. (2024)
***Conoideocrella gongyashanensis***	**China**	**Dead spider**	**-**	**Fusiform-curved,** **3.5–10.3 × 1.0–2.5**	**Yellow to light orange**	**This study**
***Metarhizium cicadae***	**China**	**Hemiptera** **(Cicada adult)**	**Cylindrical or clavate, swollen at the bottom, and gradually tapering to a thin neck, 4.2–10.1 × 1.6–3.0**	**Microconidia, ellipsoidal or ovoid, 3.6–6.8 × 2.3–3.2;** **macroconidia, cylindrical** **13.8–20.2 × 2.5–3.6**	**Bright green**	**This study**
*Metarhizium cicadae*	Thailand	Hemiptera (Cicada adult)	Cylindrical, Nomuraea-like, 4.5–10 × 2–3	Microconidia, ovoid, ellipsoidal,4–7 × 2–3.5; macroconidia, cylindrical, 10–24 × 3–4	Cream to green	Mongkolsamrit et al. (2020)
*Neoaraneomyces araneicola*	China	Spider	Single or in groups of two to three, with a cylindrical to ellipsoidal basal portion, tapering into a short distinct neck, 8.9–23.8 × 1.1–1.6	Fusiform to ellipsoidal, 2.9–4.4 × 1.3–2.0	White to pale gray	Chen et al. (2022)
***Neoaraneomyces wuyishanensis***	**China**	**Spider**	**Lanceolate, solitary, with a slender base, tapering to a thin neck, 6.2–34.3 × 0.9–2.0**	**Ellipsoidal, fusiform, and slightly curved, 2.5–4.5 × 1.0–1.4**	**White**	**This study**

Data used in this study were presented in boldface.

**Figure 3:**
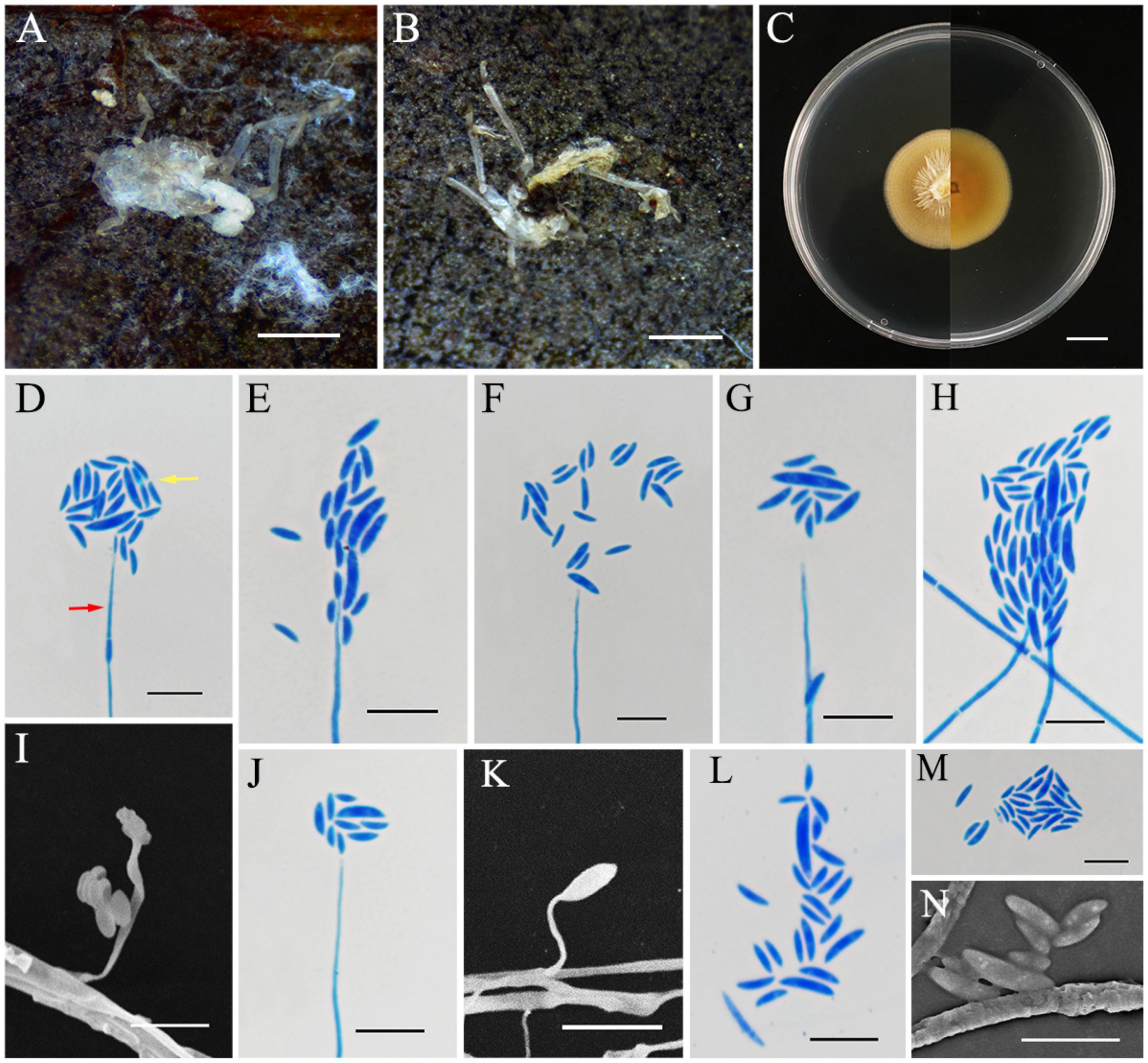
*Conoideocrella gongyashanensis* (holotype HMAS353158). **(A,B)** Dead spider infected by *C. gongyashanensis.* **(C)** Colony characteristics on PDA for 14 days. **(D–N)** Conidiogenous structures (*red arrow*) and conidia (*yellow arrow*) on PDA. Scale bars: **(A,B)** 2 mm; **(C)** 10 mm; **(D–N)**10 μm.

*Conoideocrella gongyashanensis* L. B. Lin and J. Z. Qiu, sp. nov. (Figure 3).

MycoBank No.: MB856116.

Etymology: Named after the Gongya Mountain National Forest Park, where the specimen was originally found.

Holotype: China, Fujian Province, Huaan County, Gongyashan National Forest Park. 117°25′26″ E, 24°54′15″ N, alt. 1,084 m, found on a dead spider that attaching to fallen leaves, 7 May 2024, J. Z. Qiu and L. B. Lin (HMAS353158, holotype; ex-holotype living culture CGMCC3.28305).

Sexual morph: Not observed.

Asexual morph: White to pale yellow and cottony mycelium enveloping the host. Colonies grew rapidly on PDA, reaching a diameter of 32–33 mm in 14 days at 25°C, yellow to light orange, with low mycelium density, a rigid, flocculent margin, and reverse yellowish brown. The growth rate of the colony was 23 mm/day. Hyphae were hyaline, smooth, 1.3–2.5 μm wide. The asexual phase was similar to *Hirsutella* and originated from vegetative hyphae. These featured conidiogenous structures that had a cylindrical base that gradually narrowed down toward a neck-like extension, hyaline, smooth-walled, 12.7–89.9 × 0.4–1.3 μm, mean = 53.5 × 0.9 μm. Conidia were hyaline, smooth-walled, fusiform-curved, single-celled, and clustered at the apex of the conidiogenous structures, 3.5–10.3 × 1.0–2.5 μm, mean = 6.2 × 1.59 μm.

Habitat: This species was parasitic on scale insects, found attached to the underside of fallen leaves.

Distribution: China, Fujian Province, Huaan County, Gongyashan National Forest Park.

Other materials examined: China, Fujian Province, Huaan County, Gongyashan National Forest Park. 117°25′26″ E, 24°54′15″ N, alt. 1,084 m, found on a dead spider of Sternorrhyncha that attached to the back of living leaves in May 2024, J. Z. Qiu and L. B. Lin (HMAS353159, paratype; ex-paratype living culture CGMCC3.28306).

Notes: *C. gongyashanensis* is the first species of the genus *Conoideocrella* to be discovered without an apparent sexual morph. The results of the molecular phylogenetic analysis indicate that two isolates of *C. gongyashanensis* were found which grouped together, forming a distinct branch within the genus. Phylogenetically, *C. gongyashanensis* was closely related to *C. fenshuilingensis*. Therefore, we compared the nucleotides of nr*LSU*, *tef1-α*, and *rpb1* between *C. gongyashanensis* and its related species. The differences were as follows: 1.4% (11/812, nr*LSU*), 6.7% (58/866, *tef1-α*), and 11.4% (72/630, *rpb1*) when compared to *C. fenshuilingensis*.

**Figure 4:**
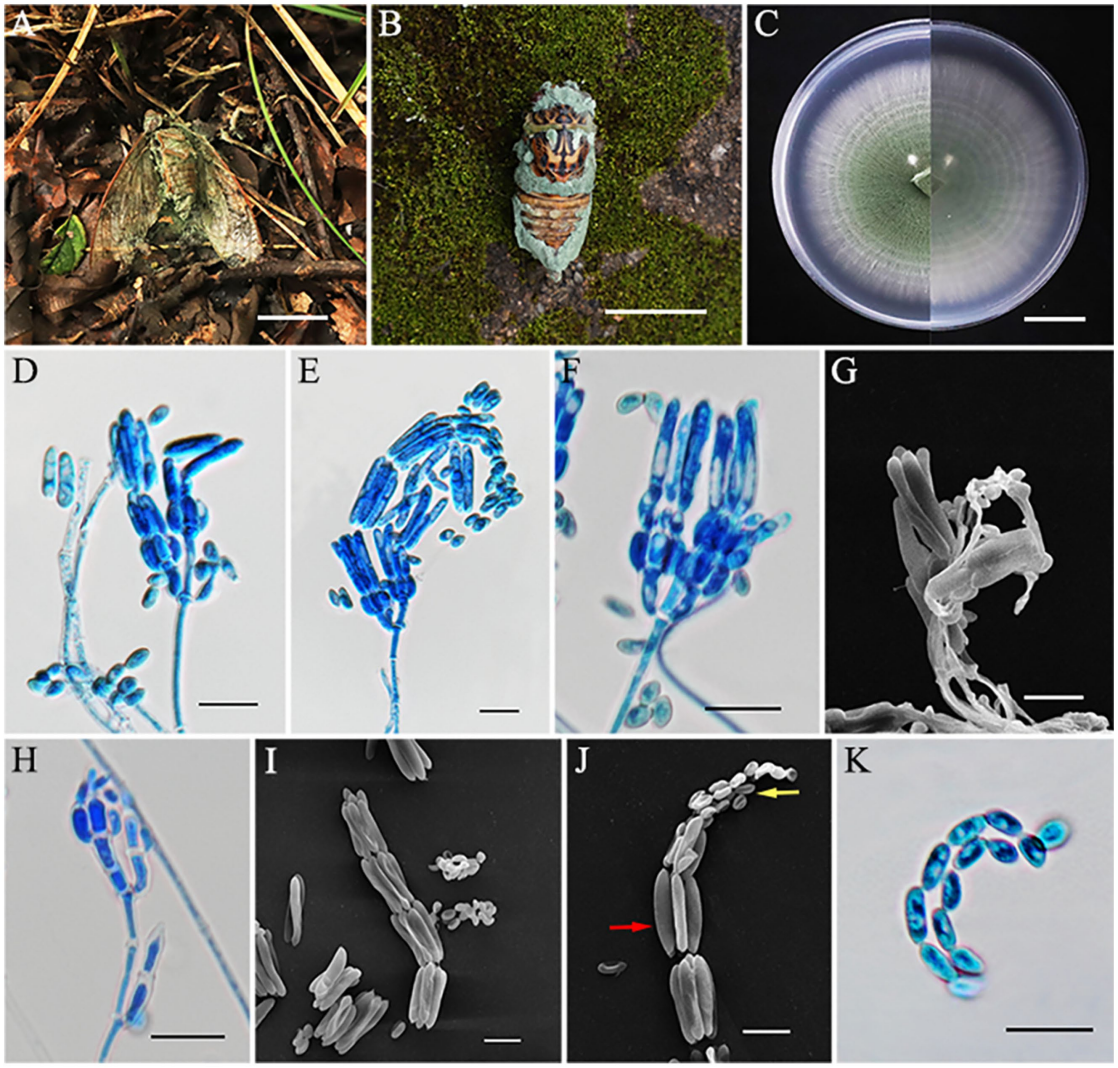
*Metarhizium cicadae* (HMAS353156). **(A,B)** Cicada adult infected by *M. cicadae.* **(C)** Colony characteristics on PDA for 14 days. **(D–H)** Part of conidiophores on PDA. **(H–I)** Microconidia (*yellow arrow*) and macroconidia (*red arrow*) on PDA. **(K)** Conidia on PDA. Scale bars: **(A,B)** 50 mm; **(C)** 20 mm; **(D–K)** 10 μm.

*Metarhizium cicadae* S. Mongkolsamrit, A. Khonsanit, D. Thanakitpipattana and J. Luangsa-ard, Stud. Mycol. 95: 212 (2020) (Figure 4).

MycoBank No.: MB856117.

Sexual morph: Not known.

Asexual morph: Cicada adult (Hemiptera) covered by densely bright green powder, produces numerous green and powdery conidia. Colonies grew rapidly on PDA and reached a diameter of 74–75 mm in 14 days at 25°C, at first white and then turning green in the center due to sporulation after 7 days. Colonies flat, floccose, with areas producing green powder, entire margin, white border, reverse as the same, with a growth rate of 5.2–5.3 mm/day. Hyphae were hyaline, branched, smooth-walled, septate, and 1.2–2.6 μm wide. Conidiophores were densely arranged, rising from aerial hyphae, with 2–3 phialides per branch. Phialides cylindrical or clavate, 4.2–10.1 × 1.6–3.0 μm, mean = 6.3 × 2.4 μm, swollen at the bottom, and gradually tapering to a thin neck. Conidia often arrayed in chains, smooth-walled, dimorphic; microconidia came first, ellipsoidal or ovoid, 3.6–6.8 × 2.3–3.2 μm, mean = 5.1 × 2.8 μm; macroconidia produced later, mostly cylindrical, 13.8–20.2 × 2.5–3.6 μm, mean = 17.5 × 3.1 μm.

Habitat: Attached to fallen leaves of dicotyledonous plants or on the trunk of trees.

Distribution: thailand (type country) and China.

Other materials examined: China, Fujian Province, Mingxi County, Gaoyang village, 116°53′38″ E, 26°33′43″ N, alt. 524 m, found on a cicada adult (Hemiptera) covered by green powder, 3 September 2023, J. Z. Qiu and L. B. Lin (HMAS353154, HMAS353155, CGMCC3.28301, CGMCC3.28302 living culture). Fujian Province, Jiangle County, Longqishan National Nature Reserve, 117°20′31″ E, 26°31′43″ N, alt. 1,275 m, found on a cicada adult (Hemiptera), 1 July 2024, J. Z. Qiu and L. B. Lin (HMAS353156, HMAS353157, CGMCC3.28303, and CGMCC3.28304 living culture).

Notes: The species *M. cicadae* was classified by Mongkolsamrit et al. in 2020, in Thailand (Luangsa-ard et al., 2017). The existence of this species has not been reported in China. In this study, four specimens were collected in Fujian Province, and, according to the phylogenetic analysis, all of them are closely related to *M. cicadae* with high statistical support (94% in ML and 0.99 in BYPP). The morphology of the four strains was generally similar to *M. cicadae*, including similarities in the host, phialides proximity, and the size of both macroconidia and microconidia (Table 3). However, the collected specimens exhibited bright green colonies on PDA, attaining 74–75 mm in 14 days, while *M. cicadae* colonies were cream to green and grew only 15 mm after 14 days of cultivation. Overall, however, our data indicated that the four strains collected in this study should be identified as *M. cicadae*.

**Figure 5:**
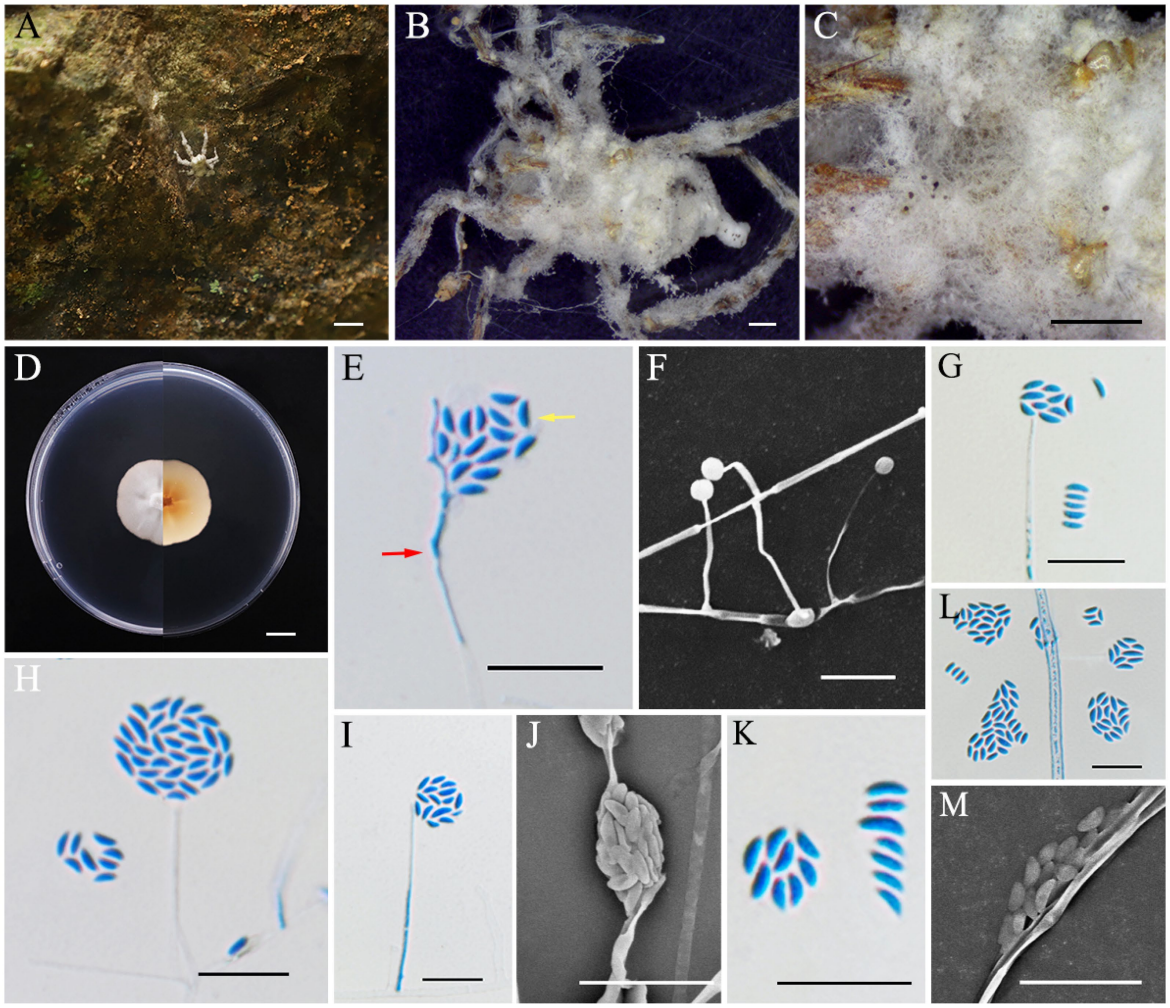
*Neoaraneomyces wuyishanensis* (holotype HMAS353160). **(A–C)** Spider infected by *N. wuyishanensis.* **(D)** Colony characteristics on PDA for 14 days. **(E–M)** Conidiophores (*red arrow*) and conidia (*yellow arrow*) on PDA. Scale bars: **(A,D)** 10 mm; **(B,C)** 1 mm; **(E–M)** 10 μm.

*Neoaraneomyces wuyishanensis* L. B. Lin and J. Z. Qiu, sp. nov. (Figure 5).

MycoBank No.: MB856118.

Etymology: Named after the Wuyi Mountain National Nature Reserve, where the species was originally found.

Holotype: China, Fujian Province, Wuyishan City, Xingcun Town, Wuyi Mountain National Nature Reserve, 117°39′12″ E, 27°36′14″ N, alt. 952 m, found on a spider adsorbed on the wall, 6 September 2023, J. Z. Qiu and L. B. Lin (HMAS353160, holotype; ex-holotype living culture CGMCC3.28307).

Sexual morph: Undetermined.

Asexual morph: The spider was completely enveloped by white to gray cottony mycelium. Colonies grew readily on PDA, showing velvety, low mycelium density, attaining a diameter of 28–30 mm after 14 days at 25°C. The colony was white, sunken slightly in the middle, reverse, light yellow to orange, with a growth rate of 2.0–2.1 mm/day. Hyphae were hyaline, smooth-walled, septate, 1.0–2.4 μm wide. Phialides were lanceolate, hyaline, originating from aerial hyphae, usually solitary, with a slender base, tapering to a thin neck, 6.2–34.3 × 0.9–2.0 μm, mean = 21.4 × 1.2 μm. Conidia were formed either at the apex of the phialides or in spherical clusters atop the phialides, smooth-walled, hyaline, ellipsoidal, fusiform and slightly curved, one-celled, 2.5–4.5 × 1.0–1.4 μm, mean = 3.1 × 1.2 μm.

Habitat: Attached to stone.

Distribution: China, Fujian Province, Wuyishan City, Xingcun Town, Wuyi Mountain National Nature Reserve.

Other materials examined: China, Fujian Province, Wuyishan City, Xingcun Town, Wuyi Mountain National Nature Reserve, 117°39′12″ E, 27°36′14″ N, alt. 952 m, found on a spider, 6 September 2023, J. Z. Qiu and L. B. Lin (HMAS353161, paratype; ex-paratype living culture CGMCC3.28308).

Notes: Multilocus analyses revealed that the two strains of *N. wuyishanensis* were similar to *N. araneicola* (DY101711, ex-holotype) with strong support (99% ML and 1 BYPP), including having the same host, parallel colony morphology, and conidia shape. The nucleotide comparison of *rpb1* and *rpb2* revealed separation from *N. wuyishanensis* and *N. araneicola* with differences of 1.67% (12/720, *rpb1*) and 1.26% (13/1031, *rpb2*), respectively. However, the arrangement of conidia between the species was quite different: the conidia of *N. wuyishanensis* sp. nov. were grouped, while those of *N. araneicola* were arranged in chains. Concurrently, the phialides of *N. wuyishanensis* sp. nov. are typically solitary, while those of *N. araneicola* are either individual or occur in small clusters of two to three. Furthermore, *N. wuyishanensis* has larger phialides (8.9–23.8 × 1.1–1.6 vs. 10.2–34.3 × 0.9–2.0 μm) and conidia (2.9–4.4 × 1.3–2.0 vs. 2.5–4.5 × 1.0–1.4 μm) compared to *N. araneicola*. In addition, after 14 days of cultivation on PDA, the mycelium of *N. wuyishanensis* sp. nov. appeared relatively fluffy, whereas that of its sibling species was slightly more compact. Moreover, this new species produced a pigment that ranged from yellow to orange (Table 3).

## 4 Discussion

The majority of insect-pathogenic fungi prefer to grow in moist, shaded areas covered with vegetation (Nasir et al., 2023). Fujian is located in the southeast of China and is known for its lush vegetation and mild climate with frequent rainfall, which makes it very suitable for both insect populations and the fungi that target them. Within the past several years, we have conducted a survey of entomogenous fungal resources in several large nature reserves and national forest parks in Fujian, during which many specimens (>50) were collected. This has led to a significant increase in the diversity of a range of fungal species (Liu et al., 2024; Mu et al., 2024; Zhao et al., 2024), highlighting the rich fungal resources of this area. In this study, we expand this diversity by characterizing of three new species of entomopathogenic fungi and one new record species, namely: *Albacillium fuzhouense*, *Conoideocrella gongyashanensis*, *Neoaraneomyces wuyishanensis*, and *Metarhizium cicadae*. All of the described species belonged to the Clavicipitaceae. This discovery not only enriches the diversity of fungal resources in the Asian continent but also sheds light on the complexity and interactions within subtropical fungal communities, which is of great significance for biological control, drug development, ecological protection, and sustainable development, and helps to unravel the evolutionary relationships and divergence patterns within subtropical fungal communities.

We identify one of the new species in this study as belonging to the genus *Albacillium*. *Albacillium* is similar to *Acremonium* and an unresolved genus *Chlorocillium* (Hypocreales incertae sedis), with *A. hingganense* reported from forest litter (but not a particular insect) in Northeast China (Ding et al., 2024), a region climatically very different from Fujian. *Albacillium* and *Chlorocillium* apparently form a monophyletic group that is basal to the Clavicipitaceae but are distinguished by conidial arrangement and other morphological features. A second species, classified within the *Conoideocrella* genus, which previously only contained four species, was identified on scale insects. *Conoideocrella,* established by Johnson et al. (2009), is similar to other genera in Clavicipitaceae, parasitizing scale insects or whiteflies (Hemiptera, Coccidae, and Lecaniidae) (Mongkolsamrit et al., 2016; Wang et al., 2024). Thus, members of *Conoideocrella* have been identified as potential specific biological control agents for (armored) scale insects, e.g., the elongate Hemlock scale, *Fiorinia externa* (Hemiptera: Diaspididae), native to Asia and which feeds on coniferous trees, and these fungi have also been exploited as potential sources of bioactive compounds including those with antimalarial and antiproliferative effects (Mongkolsamrit et al., 2016; Saepua et al., 2018; Lovett et al., 2024).

*Metarhizium* spp. are one of the most widely distributed and characterized genera of entomopathogenic fungi worldwide and are known to parasitize a wide range of insects across Hemiptera, Lepidoptera, Coleoptera, and others (Luangsa-ard et al., 2017). *Metarhizium* species are often present in soil and can form close rhizosphere associations with a broad range of plants (Chen et al., 2018a,b; Chen W. H. et al., 2018; Nishi and Sato, 2018). The genus also contains members with both broad and narrow insect host ranges that have been exploited for pest biological control (Marciano et al., 2021). Although *M. cicadae* is nominally considered potentially host (cicada)-specific, this has not been confirmed experimentally, and some caution should be taken in extrapolating from survey findings (such as this study) and definitive conclusions concerning host ranges. Given this caveat, in this study, we report a new record of the *M. cicadae* species, originally isolated from cicadas in Thailand, now found in China from an infected cicada.

Spider-pathogenic fungi are mainly distributed in the order Hypocreales, commonly found on the underside of dicotyledonous leaves, on wet cliffs, and under rocks near roadsides. Among these fungi, the genus *Gibellula* is highly regarded as a particular spider killer (Kuephadungphan et al., 2022; Joseph et al., 2024). *Neoaraneomyces* is a relatively newly described apparently arachnid-pathogen-specific genus that is related to *Claviceps*, *Hypocrella*, and *Epichloë* but distinct from a similarly characterized *Pseudometarhizium araneogenum* and currently contains only one species, *N. araneicola* (Chen et al., 2022). In this study, we have identified a second species within *Neoaraneomyces*, *N. wuyishanensis*, based on phylogenetic and morphological analyses. Little is known concerning these spider pathogens, and our data add to the diversity of potential species that either have specialized across arthropod orders and/or have significantly broad specificities.

Fujian is a region with a particularly rich diversity of fungal species (Guan et al., 2024; Liu et al., 2024). The three new species of *Albacillium*, *Conoideocrella,* and *Neoaraneomyces* collected from Fujian, as described in this study, can contribute to pest management by inhabiting pests, consuming their internal tissues, and competing with other organisms, including pests or pathogens, for resources and space. Their application in commercial pest control is promising, particularly as part of integrated pest management (IPM) strategies that align with sustainable agriculture practices, such as combining living biocontrol agents (BCAs) to improve pest control (Galli et al., 2024). Further research is required to evaluate the virulence and host specificity of these fungi under field conditions.

## Data Availability

The datasets presented in this study can be found in online repositories. The names of the repository/repositories and accession number(s) can be found in the article/Supplementary material.

## Footnotes

^1^ https://itol.embl.de

## Supplementary material

SUPPLEMENTARY FIGURE 1 Sampling sites map of Fujian Province, China. Notes: In the map, FAFU, GYC, GYS, LQS, and WYS correspond to Fuzhou City, Gaoyang Village, Gongyashan National Forest Park, Longqishan National Nature Reserve, and Wuyi Mountain National Nature Reserve, respectively.
