# Supplementary figures and images for: New entomopathogenic species in the Clavicipitaceae family (Hypocreales, Ascomycota) from the subtropical forests of Fujian, China
Source: Front Microbiol. 2025 Mar 3;16:1532341. doi: 10.3389/fmicb.2025.1532341 (PMC11911381; doi:10.3389/fmicb.2025.1532341)

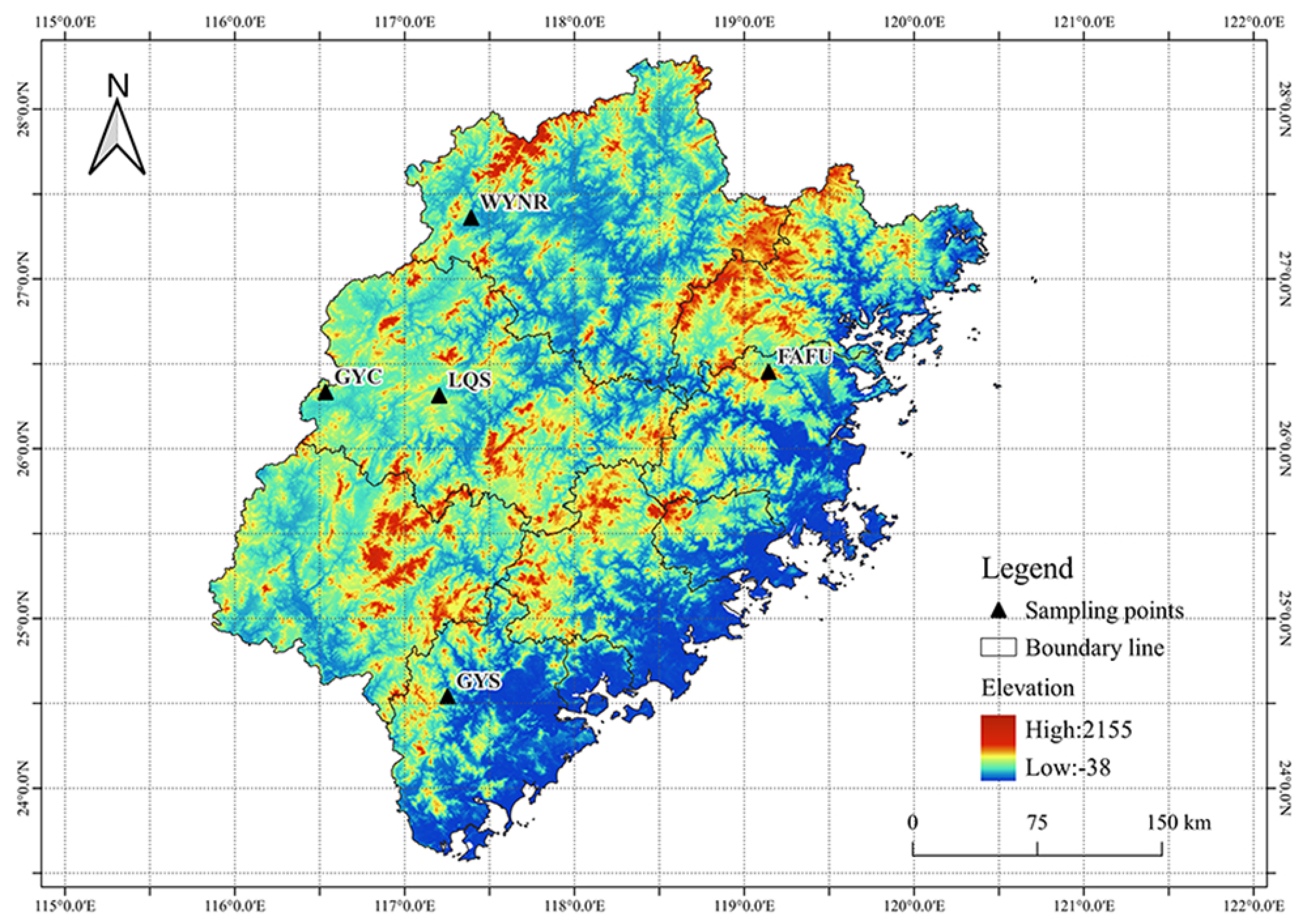

Supplement: SUPPLEMENTARY FIGURE 1 — Sampling sites map of Fujian Province, China. Notes: In the map, FAFU, GYC, GYS, LQS, and WYS correspond to Fuzhou City, Gaoyang Village, Gongyashan National Forest Park, Longqishan National Nature Reserve, and Wuyi Mountain National Nature Reserve, respectively. [file Image_1.JPEG]
